# Analysis of Hub Genes Involved in Distinction Between Aged and Fetal Bone Marrow Mesenchymal Stem Cells by Robust Rank Aggregation and Multiple Functional Annotation Methods

**DOI:** 10.3389/fgene.2020.573877

**Published:** 2020-12-14

**Authors:** Xiaoyao Liu, Mingjing Yin, Xinpeng Liu, Junlong Da, Kai Zhang, Xinjian Zhang, Lixue Liu, Jianqun Wang, Han Jin, Zhongshuang Liu, Bin Zhang, Ying Li

**Affiliations:** ^1^Institute of Hard Tissue Development and Regeneration, The Second Affiliated Hospital of Harbin Medical University, Harbin, China; ^2^Heilongjiang Academy of Medical Sciences, Harbin, China

**Keywords:** bone marrow mesenchymal stem cell, fetal stem cell, adult setm cell, robust rank aggregation, hub genes

## Abstract

Stem cells from fetal tissue protect against aging and possess greater proliferative capacity than their adult counterparts. These cells can more readily expand *in vitro* and senesce later in culture. However, the underlying molecular mechanisms for these differences are still not fully understood. In this study, we used a robust rank aggregation (RRA) method to discover robust differentially expressed genes (DEGs) between fetal bone marrow mesenchymal stem cells (fMSCs) and aged adult bone marrow mesenchymal stem cells (aMSCs). Multiple methods, including gene set enrichment analysis (GSEA), Gene Ontology (GO) analysis, and Kyoto Encyclopedia of Genes and Genomes (KEGG) pathway analysis were performed for functional annotation of the robust DEGs, and the results were visualized using the R software. The hub genes and other genes with which they interacted directly were detected by protein–protein interaction (PPI) network analysis. Correlation of gene expression was measured by Pearson correlation coefficient. A total of 388 up-regulated and 289 down-regulated DEGs were identified between aMSCs and fMSCs. We found that the down-regulated genes were mainly involved in the cell cycle, telomerase activity, and stem cell proliferation. The up-regulated DEGs were associated with cell adhesion molecules, extracellular matrix (ECM)–receptor interactions, and the immune response. We screened out four hub genes, *MYC*, *KIF20A*, *HLA-DRA*, and *HLA-DPA1*, through PPI-network analysis. The *MYC* gene was negatively correlated with *TXNIP*, an age-related gene, and *KIF20A* was extensively involved in the cell cycle. The results suggested that MSCs derived from the bone marrow of an elderly donor present a pro-inflammatory phenotype compared with that of fMSCs, and the *HLA-DRA* and *HLA-DPA1* genes are related to the immune response. These findings provide new insights into the differences between aMSCs and fMSCs and may suggest novel strategies for *ex vivo* expansion and application of adult MSCs.

## Introduction

Stem cells can be isolated at all stages of ontogeny, from the early developing embryo to the post-reproductive adult organism. Adult stem cells are less potent than embryonic stem cells, but still play a very important role in maintaining overall health (Jin, 2017). Adult bone marrow was the first source of mesenchymal stem cells (MSCs) to be identified and is still by far the best characterized (Friedenstein et al., 1987; Kolf et al., 2007; Lv et al., 2014). These cells hold great promise as seed cells in tissue engineering and regenerative medicine, based on their self-renewal, multi-differentiation, and immunoregulation abilities (Dogan et al., 2014; Wei et al., 2015; Castagnini et al., 2016; Mehrabani et al., 2016). However, there is growing evidence that demonstrates that the number of bone marrow-derived MSCs is limited and declines with the age of the donor (Dexheimer et al., 2011). Thus, long-term cell culture is needed to obtain large numbers of cells suitable for clinical applications. However, MSCs may undergo senescence, as well as impaired function during *ex vivo* expansion (Turinetto et al., 2016).

Although fetal and adult MSCs share the same morphology and surface molecules, previous studies have shown that MSCs from fetal tissues are more adaptable, with greater self-renewal capacity, both *in vivo* and *in vitro* (O’Donoghue and Fisk, 2004; Guillot et al., 2007; Ding et al., 2011). The prevalence of MSCs in fetal bone marrow is significantly higher than that in adult tissue (O’Donoghue and Fisk, 2004; Ding et al., 2011). Fetal MSCs are readily expandable *in vitro*, with a shorter doubling time, and display no obvious change in phenotype after 20 passages (Campagnoli et al., 2001). Existing research recognizes the critical role telomerase plays in self-renewal and the replicative potential of stem cells (Hayflick, 2000). Comparative studies of fetal liver hematopoietic stem cells (HSCs) and adult bone marrow HSCs have confirmed that fetal stem cells have higher telomerase activity and again suggest that proliferative potential is limited and declines with age (Verfaillie et al., 2002). In addition, telomere length is longer in fetal MSCs (Xu et al., 2016).

Another advantage of MSCs is their immunomodulatory properties (Chen et al., 2011; Andrzejewska et al., 2019). Bone marrow MSCs (BM-MSCs) from both adults and fetuses reportedly possess immune-suppressive effects. Fetal MSCs display immunological inertness and appear to have stronger immunomodulatory abilities than their adult counterparts (Götherström et al., 2005; Chang et al., 2006).

Mesenchymal stem cells isolated from fetal tissue may therefore have greater potential for clinical application, but the exact mechanisms by which they exert their effects are still not very clear. Moreover, the application of fetal tissue is not widely accepted and is still being debated. Understanding the difference between fetal and adult stem cells and their regulatory mechanisms may provide new insights for the clinical application of adult stem cells.

In this study, two existing datasets from Gene Expression Omnibus (GEO) were analyzed by the robust rank aggregation (RRA) method, which facilitates the detection of genes that are ranked consistently in multiple datasets and assigns a significance score for each gene (Reimand et al., 2010). This method was used to identify robust differentially expressed genes (DEGs) between MSCs derived from elderly adult bone marrow and fetal bone marrow. Functions of these robust DEGs were then explored by gene set enrichment analysis (GSEA), Gene Ontology (GO), and Kyoto Encyclopedia of Genes and Genomes (KEGG) analyses. Using protein–protein interaction (PPI) network analysis, we screened out four hub genes, *MYC*, *KIF20A*, *HLA-DRA*, and *HLA-DPA1*, which were closely related to function. Furthermore, GO and KEGG enrichment analyses were utilized to verify potential biological functions of hub genes and their first neighbors.

## Materials and Methods

### Microarray Data Information

All available datasets were acquired from the GEO database^1^. The screening criteria of datasets were inclusion of gene expression data of BM-MSCs from fetal and aged donors. Eventually, two datasets, GSE97311 (Spitzhorn et al., 2019) and GSE68374 (Paciejewska et al., 2016), were included in the study. Series matrix files and platform information of GSE97311 and GSE68374 were downloaded from the GEO database for further study. The GSE97311 dataset contained three fetal femur-derived MSC samples and four adult MSC samples. The GSE68374 dataset included three biological replicates for both fetal and adult bone marrow-derived MSC samples.

### Data Processing and Identification of Robust DEGs

The microarray data of GSE97311 and GSE68374 were initially normalized and differential expression was analyzed using the R software though the “limma” package (Ritchie et al., 2015). The results were presented on a volcano plot (Li, 2012). We then used the “RobustRankAggreg” package (Reimand et al., 2010) to integrate the differential expression results of the two datasets to identify the robust DEGs. As this RRA method screens genes ranked consistently better than expected based on null hypothesis of uncorrelated inputs, batch effect correction is not needed (Liu et al., 2018). Benjamini–Hochberg’s method was used to control the false discovery rate (FDR). The *P*-value of each gene represents its ranking in the final gene list. Genes with a *P*-value < 0.05 and | logFC| > 1 in the final list were considered significant DEGs for the next mining. The R package “pheatmap” was used to visualize expression patterns of the top 40 DEGS (top 20 up-regulated genes and top 20 down-regulated genes) from RRA analysis.

### Gene Set Enrichment Analysis (GSEA)

The following sets were downloaded from the Molecular Signatures Database version 7.1^2^ : H.all.v7.symbles.gmt, c2.cp.kegg.v7.1.symbols.gmt, and other interesting gene sets involved in the oxidative response, production of interleukin 6, telomerase activity, and stem cell self-renewal in c5.bp.v7.1.symbols.gmt. The GseaPreranked tool was then used to perform enrichment analysis for all the DEGs integrated via RRA method, which are ranked by log FC from large to small. Gene set permutations were performed 1000 times for each analysis. We then visualized the results of GSEA using “ggplot2” in the R package (Ito and Murphy, 2013).

### Functional Enrichment Analysis of Robust DEGs

BinGO (Maere et al., 2005), a plug-in of Cytoscape, was used for GO enrichment. The KEGG pathway analyses were conducted by the R package “clusterprofiler” (Yu et al., 2012). The GO terms and KEGG pathways with adjusted *P*-value < 0.05 were considered statistically significant and visualized by the “GOplot” package (Walter et al., 2015). The Z-score was calculated, which hinted at whether the biological process (or/molecular function/cellular component) or KEGG pathway was more likely to be reduced (negative value) or increased (positive value).

$$z-score=\backslash frac\left\{\left(up-down\right)\right\}\left\{\sqrt{\left\{c\,o\,u\,n\,t\right\}}\right\}$$

### Identification of Hub Genes and Their First Neighbors by PPI Network Analysis

The DEGs with *P* < 0.001 were defined as the most robust DEGs and uploaded to the STRING database to establish a PPI network (Szklarczyk et al., 2017). Interaction scores > 0.4 were set as the cut-off point. The STRING analysis results were then imported into the Cytoscape software version 3.8.0, and the network was ranked by degree and betweenness methods using the cytoHubba plug-in (Chin et al., 2014) to select hub genes. Hub genes were screened according to the degree score > 10 and ranked at the top 10 of total genes, sorted by the betweenness method. We then selected the first neighbors of hub genes that were directly related to the hub genes, to construct their sub-networks, respectively.

### Correlation Analysis and Functional Enrichment Analysis

Correlations were analyzed by Pearson’s correlation (Schober et al., 2018) for genes involved in the sub-network, which was built by hub gene and its first neighbors. Genes with a Pearson’s correlation coefficient greater than 0.5 were considered most significant correlation with hub genes and were selected for GO annotation and KEGG pathway enrichment analysis. A total of 13 samples were included in correlation analysis and the expression of genes was obtained from GSE97311 and GSE68374 datasets.

## Results

### Identification of Robust DEGs by the RRA Method

**FIGURE 1:**
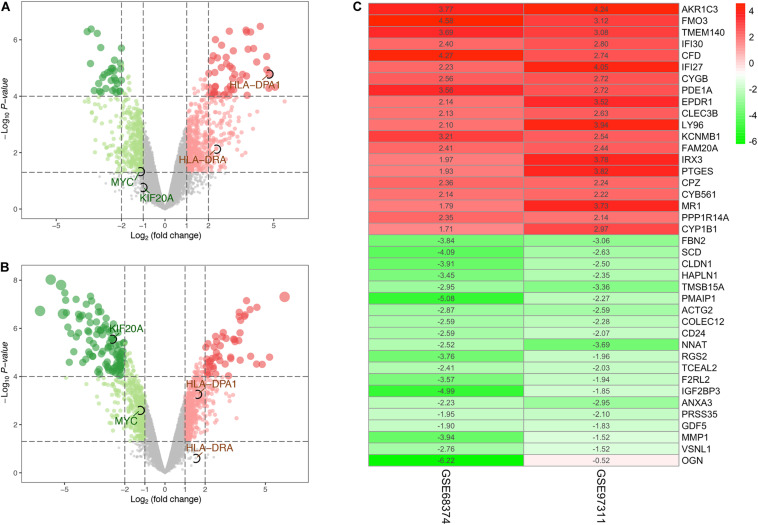
Identification of differentially expressed genes (DEGs). **(A)** Volcano plot of DEGs for dataset GSE68374. **(B)** Volcano plot of DEGs for dataset GSE97311. **(C)** The heatmap of the top 40 (20 up-regulated and 20 down-regulated) DEGs according to *P*-value identified by the RRA method. The number in the heat map represents log FC. The gradual color ranging from red to green indicates the changing process from up- to down-regulation. Genes circle by black ellipses are the hub genes screened out by subsequent analysis.

The expression profiles of aged adult BM-MSCs (aMSCs) were compared with those of fetal BM-MSCs (fMSCs). Based on the screening criteria of *P* < 0.05 and | logFC| > 1, a total of 933 DEGs were identified from GSE97311, including 553 up-regulated genes and 380 down-regulated genes (Figure 1A). In addition, 993 DEGs, including 496 up-regulated genes and 497 down-regulated genes, were identified from GSE68374, according to the same criteria (Figure 1B). We integrated the results of the two datasets using the RRA method, and obtained 14,024 up-regulated genes and 9872 down-regulated genes (Supplementary Table S1), which finally yielded 677 robust DEGs. A heatmap of the top 20 up-regulated robust genes and top 20 down-regulated robust genes are presented in Figure 1C, and a complete list of the robust DEGs is provided in Supplementary Table S2. The most significant up-regulated gene was *AKR1C3* (*P* = 4.24E-07, logFC = 4.005), followed by *FMO3* (*P* = 4.79E-06, logFC = 3.849), and *TMEM140* (*P* = 5.60E-06, logFC = 3.385). Moreover, *FBN* (*P* = 2.02E-06, logFC = −3.449); *SCD* (*P* = 4.05E-06, logFC = −3.363); *CLDN1* (*P* = 5.60E-06, logFC = −3.209) were the most significant down-regulated genes.

### GSEA Reveals Differences Between aMSCs and fMSCs

**FIGURE 2:**
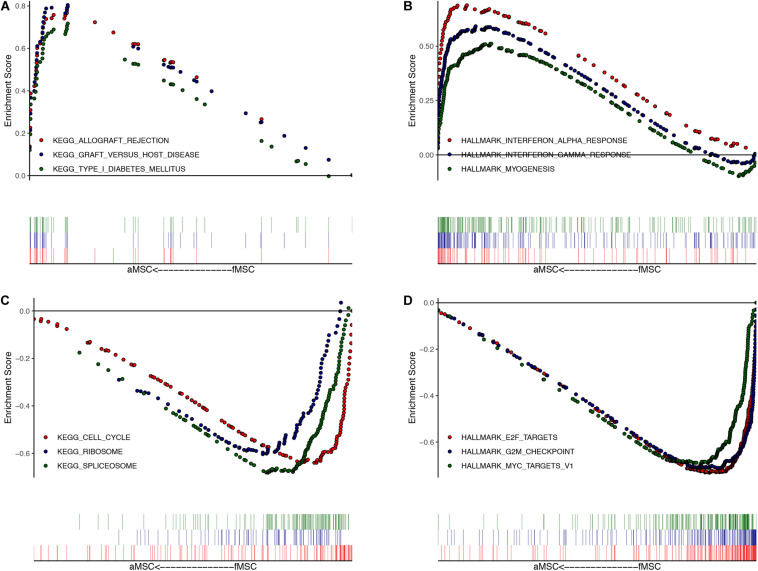
Gene set enrichment analysis (GSEA) for DEGs. The top three enriched gene sets (according to GSEA enrichment score) in aMSC group: **(A)** for KEGG; **(B)** for HALLMARK. The top three enriched gene sets (according to GSEA enrichment score) in fMSC group: **(C)** for KEGG; **(D)** for HALLMARK. All gene sets are significantly enriched at a false discovery rate (FDR) < 0.05. The location of the vertical bar shows the occurrence of the gene in the ranked list.

The ranked list of DEGs is shown in Supplementary Table S3. Results of GSEA for KEGG pathways revealed that most up-regulated DEGs in aMSCs were related to immune-related diseases (Figure 2A), and down-regulated DEGs were abundant in the cell cycle, ribosome pathway, and spliceosome pathway (Figure 2C). Using hallmark gene sets, the interferon response and myogenesis were enriched in aMSCs (Figure 2B). Moreover, E2F targets, the G2M checkpoint, and MYC targets were enriched in fMSCs (Figure 2D). Gene sets with the highest enrichment scores were all associated with the cell cycle. All gene sets were significantly enriched at an FDR < 0.05.

### Analysis of Gene Sets Related to Aging and Stem Cell Self-Renewal

**FIGURE 3:**
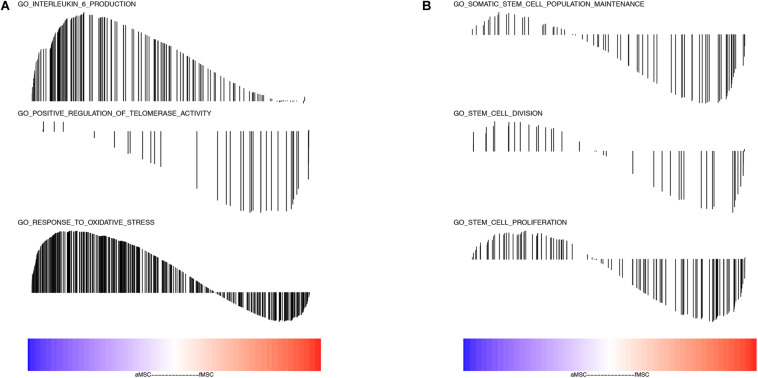
Analyses of gene sets related to aging and stem cell self-renewal. **(A)** Interleukin 6 production (NES = 1.939, FDR q-value = 0.019); positive regulation of telomerase activity (NES = –1.616, FDR q-value = 0.021); response to oxidative stress (NES = 1.4371426, FDR q-value = 0.076). **(B)** Somatic stem cell population maintenance (NES = –1.378, FDR q-value = 0.069); stem cell division (NES = –1.207, FDR q-value = 0.158); stem cell proliferation (NES = –1.266, FDR q-value = 0.046). The color bar indicates the sort order of genes (blue represents up-regulated in aMSC group and red represents up-regulated in fMSC group). The location of vertical bar indicates the occurrence of that gene within the ranked list and the height of the bar indicated the enrichment score.

To further determine the functions of robust DEGs in specific biological processes, we performed GSEA analysis related to aging and stem cell self-renewal. The GO annotation in terms of response to oxidative stress and IL-6 production enriched in the aMSC group (Figure 3A). The GO gene sets of telomerase activity, somatic stem cell population maintenance, stem cell division, and stem cell proliferation were all enriched in fMSCs (Figure 3B).

### GO and KEGG Enrichment Analysis of Robust DEGs

**FIGURE 4:**
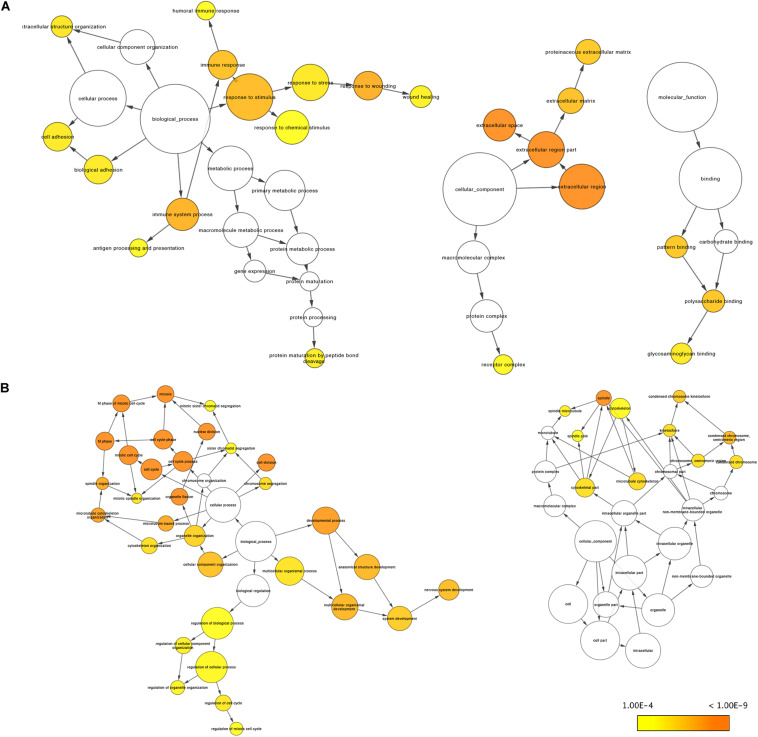
Gene Ontology analyses of robust up-regulated **(A)** and down-regulated **(B)** DEGs by BINGO plug-in. There is no enrichment for molecular function in **B**. The size of the nodes is proportional to the number of genes in that term. Significance decreases from dark orange (*p* = 1.00E-9) to yellow (*p* = 1.00E-4).

**FIGURE 5:**
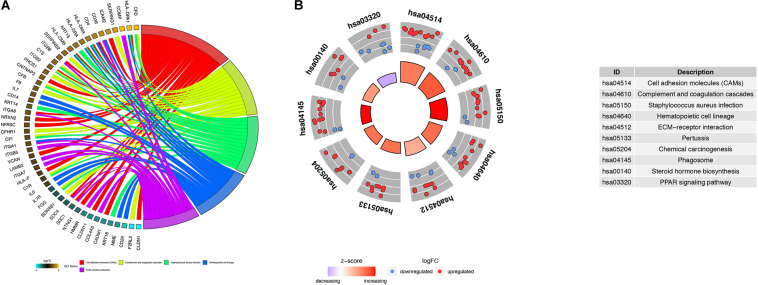
Kyoto Encyclopedia of Genes and Genomes (KEGG) analysis of robust DEGs. Chord plot **(A)** and circle plot **(B)** show the relationship between genes and KEGG pathways. **(A)** Chord plot depicting the relationship between genes and the top five KEGG pathways. **(B)** Circle plot depicting the distribution of genes in the top 10 KEGG pathways.

Using the BINGO plug-in, we obtained a global perspective of the changes in gene expression patterns. The up-regulated DEGs in aMSCs were enriched in biological processes such as the immune response, response to stimulus, extracellular structure organization, cell adhesion, and biological adhesion (Figure 4A). In contrast, down-regulated DEGs were mainly enriched in the cell cycle and developmental processes (Figure 4B). Based on the results of KEGG enrichment analysis, the top five significant pathways were cell adhesion molecules, complement and coagulation cascades, *Staphylococcus aureus* infection, hematopoietic cell lineage, and extracellular matrix (ECM)–receptor interaction. Unlike the other pathways, the peroxisome proliferator-activated receptor (PPAR) signaling pathway was more likely to be inhibited in aMSCs (Figures 5A,B).

### Identification of Hub Genes and Their First Neighbors

**FIGURE 6:**
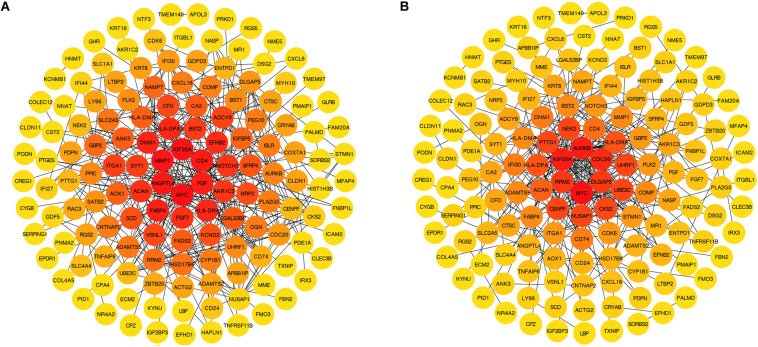
DEGs with a *P*-value < 0.001 ranked by betweenness **(A)** and degree **(B)** methods using cytoHubba in Cytoscape. The change of color from orange red to yellow represents the change of centrality score from high to low.

**FIGURE 7:**
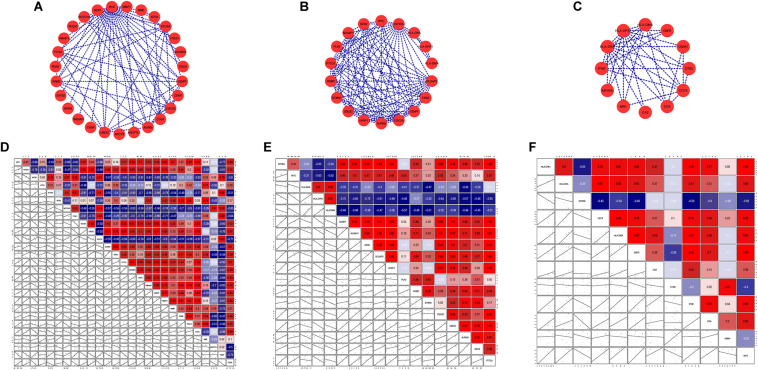
Sub-networks **(A–C)** and correlation matrixes **(D–F)** of hub genes and their first neighbors: **(A,D)** *MYC*; **(B,E)** *KIF20A*; **(C,F)** *HLA-DRA* and *HLA-DPA1*.

The PPI network established by the most robust DEGs (*P* < 0.001) contained 144 nodes and 291 edges. We screened four hub genes, considering the degree (DC) and betweenness (BC) centrality (Figures 6A,B). Among them, *MYC* (DC = 26, BC = 9816.96488) and *KIF20A* (DC = 17, BC = 4690.54753) were up-regulated in the fMSC group, and *HLA-DRA* (DC = 11, BC = 2105.09486) and *HLA-DPA1* (DC = 11, BC = 2105.09486) were up-regulated in the aMSC group. We then selected their first neighbors and structured the respective sub-networks. As shown in Figures 7A–C, there were 26 nodes and 63 edges in the sub-network of *MYC*; and 18 nodes and 89 edges in the sub-network of *KIF20A*. Both *HLA-DRA* and *HLA-DPA1* were part of the same network, which included 12 nodes and 34 edges.

### Correlation Analysis and Functional Enrichment Analysis of Sub-Networks

**FIGURE 8:**
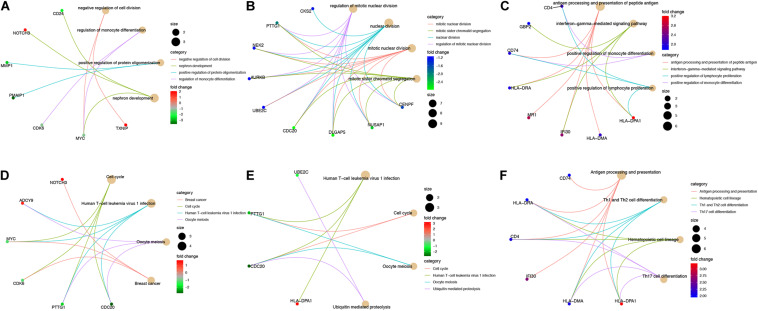
Interactive genes of hub-genes were filtered by a Pearson correlation coefficient > 0.5 and GO **(A–C)** and KEGG pathway **(D–E)** analyses were performed for these genes. GO and KEGG analyses for **(A,D)** interactive genes of *MYC*; **(B,E)** interactive genes of *KIF20A*; **(C,F)** interactive genes of *HLA-DRA* and *HLA-DPA1*. Cut off = adjust *P* < 0.05, the top four are shown in plot.

We analyzed gene-gene expression correlation coefficients for genes in sub-networks (Figures 7D–F) and filtered out genes with a Pearson correlation coefficient > 0.5. Correlation coefficient, *P*-values, and coefficient of variation for all the genes included in the correlation analysis are shown in Supplementary Tables S4, S5. Some interesting examples are shown in Supplementary Figure S1. The GO and KEGG pathway analyses were also performed for these genes (Figures 8A–F). For *MYC*, we observed that both *MYC* and *TXNIP*, a gene up-regulated in aMSCs, were involved in negative regulation of cell division. The KEGG analysis showed that the cell cycle, breast cancer, oocyte meiosis, and human T-cell leukemia virus 1 infection were enriched. Several biological processes and GO terms, such as miotic nuclear division, mitotic sister chromatid segregation, and nuclear division, were abundant in *KIF20A*-related genes. Pathways implicated with these genes were similar to *MYC* and its closely related neighbors. For *HLA-DRA* and *HLA-DPA1*, GO terms such as antigen processing and presentation of peptide antigen, and the interferon gamma mediated signaling pathway were enriched; and KEGG pathways associated with antigen processing and presentation, hematopoietic cell lineage, and Th cell differentiation were enriched.

## Discussion

Human BM-MSCs (hBM-MSCs) are promising sources for tissue engineering and regenerative medicine. Human fetal MSCs (hf-MSCs) have more primitive expression profiles and greater proliferative capacity than their adult counterparts (Spitzhorn et al., 2019). These cells can more readily expand *in vitro* and senesce later in culture. Both aMSCs and fMSCs harbor immunomodulatory capacity and are non-immunogenic, even though some differences have been reported (Götherström et al., 2005; Chang et al., 2006; Chen et al., 2011; Andrzejewska et al., 2019). The underlying molecular mechanisms for these differences are still not fully understood.

In this study, we used bioinformatics to mine the underlying molecular mechanisms that explain the difference between aMSCs and fMSCs. To our best knowledge, this is the first study to use the RRA method to analyze the difference between aMSC and fMSC sources in human bone marrow. Götherström et al. (2005) explored the gene expression profile of MSCs derived from the fetal liver and adult bone marrow. Other studies have shown that MSCs derived from different sources possess distinct biological properties (Berebichez-Fridman and Montero-Olvera, 2018; Kozlowska et al., 2019).

Only two datasets in the GEO database, GSE97311 and GSE 68374, met our experimental requirements. We performed robust differential expression profiling analysis using the two existing GEO datasets and obtained 677 robust DEGs, including 388 up-regulated and 289 down-regulated DEGs in aMSCs compared with fMSCs. The most significantly up-regulated gene in aMSCs was *AKR1C3*. This gene may play an important role in the pathogenesis of allergic diseases such asthma and may have a role in controlling cell growth or differentiation. The most significantly up-regulated gene in fMSCs was *FBN2*, which regulates the early processes of elastic fiber assembly and osteoblast maturation.

The GSEA was conducted for all DEGs in the final robust rank list. Gene sets abundant in fMSCs, such as those associated with the cell cycle, E2F targets, and *MYC* targets, were all related to proliferation. These results further support the notion that fMSCs have greater self-renewal abilities than aMSCs and are consistent with earlier observations (Chen et al., 2011). One unexpected finding was the up-regulated immune response in aMSCs. We found all of the most significantly enriched gene sets in aMSCs involved in the immune response. Several reports have shown that aging leads to the pro-inflammatory phenotype, with activated innate immune responses (Danilova, 2006; Salminen et al., 2008). The chronic low-grade inflammatory state of elderly donors may be the reason for the heightened immune response of aMSCs.

As previous studies have shown, IL-6 is a reliable aging parameter and senescent MSCs release excess IL-6 (Salminen et al., 2008; Suvakov et al., 2019). Thus, we conducted GSEA on interesting biological processes, including IL-6 production, telomerase activity, oxidative stress, and stem cell self-renewal. The results showed enrichment of IL-6 production and oxidative stress in aMSCs; and enrichment of telomerase activity and stem cell proliferation related gene sets in fMSCs. These results further suggest that MSCs derived from elderly adults possess age-related characteristics. The disadvantages of aMSCs could be partially attributed to these intrinsic age-related drawbacks.

Consistent with published data, robust DEGs enriched in several KEGG pathways, such as cell adhesion and ECM-receptor, which also participate in the immune response, are reportedly down-regulated pluripotency markers that inhibit mouse embryonic stem cell self-renewal (Taleahmad et al., 2015). The PPAR pathway is down-regulated in aMSCs; and this might be explained by reduced PPAR activity related to increased inflammation levels in old age (Michalik and Wahli, 2008). Regarding GO annotation, the up-regulated DEGs in adults compared with those in fetuses were involved in the immune response, and cell–cell and cell–ECM contact; whereas down-regulated expression was observed in aMSCs compared with fMSCs in cell cycle progression and development.

The PPI network was then constructed by the most robust DEGs with *P*-values < 0.001 and |logFC| > 1, to evaluate the relationship between these genes and identify hub genes. We detected four hub genes: *MYC*, *KIF20A*, *HLA-DRA*, and *HLA-DPA1* according to BC and DC.

Prior studies have noted the important role of *MYC* in a range of cellular processes, including proliferation, the cell cycle, and pluripotency maintenance in stem cells (Lüscher, 2001; Kumamoto et al., 2009; Chen et al., 2018). Furthermore, c-MYC can inhibit replicative senescence caused by telomere damage by promoting the expression of human telomerase reverse transcriptase (hTERT), a catalytic subunit of telomerase (Xu et al., 2001). Past research has revealed that high expression of c-MYC is associated with increased self-renewal and differentiation, which is regulated by Sox2 (Park et al., 2012). A recent study showed that the proliferative capacity of human MSCs derived from bone marrow is linked to c-MYC expression (Melnik et al., 2019). Sufficient c-MYC expression may be essential to maintain high proliferative rates and an undifferentiated state of MSCs during *ex vivo* expansion.

In the present study, *MYC* as a hub gene was up-regulated in fetal BM-MSCs. We found a negative correlation between the expression of *MYC* and *TXNIP* (correlation coefficient = −0.77). Oberacker et al. (2018) showed that *TXNIP* plays a crucial role in aging processes. Age-related up-regulation of *TXNIP* results in reduced resistance to oxidative stress, and thereby accelerates aging. The present study reveals that fMSCs with high expression of *MYC* and low expression *TXNIP* may explain why fMSCs possess greater proliferative capacity and are more resistant to aging. The negative correlation between *MYC* and *TXNIP* in BM-MSCs warrants further research. Nevertheless, we must also acknowledge that high levels of *c-MYC* increase the risk of oncogenesis (Kozlowska et al., 2019).

The mitotic kinesin, *KIF20A*, is essential for central spindle organization at anaphase as well as cytokinesis regulation (Zhang et al., 2019). It is supposedly a key factor in cell proliferation and invasion in many cancers (Sheng et al., 2018; Zhang et al., 2019). A recent study showed that *KIF20A* could reportedly be regulated by *MYC* (Pan et al., 2020). There is currently no data regarding the function of *KIF20A* in adult stem cells, and the relationship between *MYC* and *KIF20A* is unclear. Our findings suggest that *KIF20A* is extensively linked with the cell cycle in BM-MSCs, and is moderately correlated (correlation coefficient = 0.41) with *MYC*.

Another major difference was observed in immunoregulatory function. Both *HLA-DRA* and *HLA-DPA1* belong to the major histocompatibility complex class II, are up-regulated in aMSCs, and mainly involved in antigen procession and presentation. The aMSCs express intermediate levels of HLA class **I** and low levels of HLA class **II**, while fMSCs express no HLA class **II** (Gotherstrom et al., 2003; O’Donoghue and Fisk, 2004). Previous reports have shown that adult MSCs contain intracellular deposits of class **II** alloantigen, and their surface expression can be induced under inflammatory conditions, such as in the presence of INFγ (Gotherstrom et al., 2003; Ryan et al., 2005). The BM-MSCs can therefore be recognized by allogeneic lymphocytes, possess immunomodulatory properties *in vitro*, and suppress the proliferation of activated lymphocytes (Ryan et al., 2005; Chen et al., 2011). However, Gallipeau and colleagues proposed discrepancies in the immune-suppressive activities of MSCs arising from intrinsic variability of each donor source, with an average age > 65 years (Romieu-Mourez et al., 2012). Furthermore, the immunomodulation of MSCs can be regulated by inflammatory conditions; in low-level inflammatory microenvironments, BM-MSCs promote inflammation and act as antigen-presenting cells (Betancourt, 2013). The BM-MSCs from elderly donors in this study seemed to have a pro-inflammatory phenotype, which may be due to the chronic low-grade inflammatory conditions of aged donors.

As for cancerous condition, there is a growing body of evidence suggests that it plays a key role in the maintenance and progression of tumor. Fernando et al. highlight the importance of tumor microenvironment, especially for MSC, in multiple myeloma (MM) (Fernando et al., 2019). The telomeric length of MM – MSC is more lower and genes, such as CDC20, CDC6, involved in cell cycle are decrease in expression, which exhibit similar down-regulated in aMSCs. However, immune response related genes, for instance, HLA-DRA, are also down-regulated in MM – MSC, which is up-regulated in aMSCs.

## Conclusion

Through data mining and network analysis, we detected four hub genes, *MYC*, *KIF20A*, *HLA-DRA*, and *HLA-DPA1*. Expression of the *MYC* gene was negatively correlated with that of *TXNIP*, a known senescence-associated gene. Furthermore, *KIF20A* is extensively linked with the cell cycle. The other two core genes, *HLA-DRA* and *HLA-DPA1*, are implicated in the immune response and may be induced by age-related inflammatory conditions. We infer that BM-MSCs derived from elderly donors may have age-related drawbacks. These cells show lower proliferative capacity and a pro-inflammatory phenotype. More experiments are required for further verification of these findings.

## Data Availability Statement

The original contributions presented in the study are included in the article/Supplementary Material. Further inquiries can be directed to the corresponding author/s.

## Author Contributions

XYL conceived and designed the research, performed analysis, and wrote the majority of the manuscript. BZ, YL, HJ, and ZL supervised the research. MY, XPL, JD, KZ, XZ, LL, and JW contributed to data collation. All authors read and approved the final manuscript.

## Conflict of Interest

The authors declare that the research was conducted in the absence of any commercial or financial relationships that could be construed as a potential conflict of interest.

## Footnotes

1 www.ncbi.nlm.nih.gov/geo/

2 www.gsea-msigdb.org/gsea/msigdb/index.jsp

## Supplementary Material

Supplementary Figure 1 The correlation profiles of **(A)** *MYC* and *TXNIP*; **(B)** *KIF20A* and *CDC20*; **(C)** *HLA*-*DPA1* and *MR1*; **(D)** *HLA-DRA* and *CD74*.

Supplementary Table 1 The full list of up- and down-regulated genes integrated by RRA method.

Supplementary Table 2 The details of all the robust DEGs.

Supplementary Table 3 The list of DEGs ranked by log FC from large to small.

Supplementary Table 4 Correlation coefficient and *P*-values for the correlation between genes in sub-network and hub genes.

Supplementary Table 5 Coefficient of variation for all the genes included in the correlation analysis.
