# Supplementary figures and images for: Analysis of Hub Genes Involved in Distinction Between Aged and Fetal Bone Marrow Mesenchymal Stem Cells by Robust Rank Aggregation and Multiple Functional Annotation Methods
Source: Front Genet. 2020 Dec 14;11:573877. doi: 10.3389/fgene.2020.573877 (PMC7793715; doi:10.3389/fgene.2020.573877)

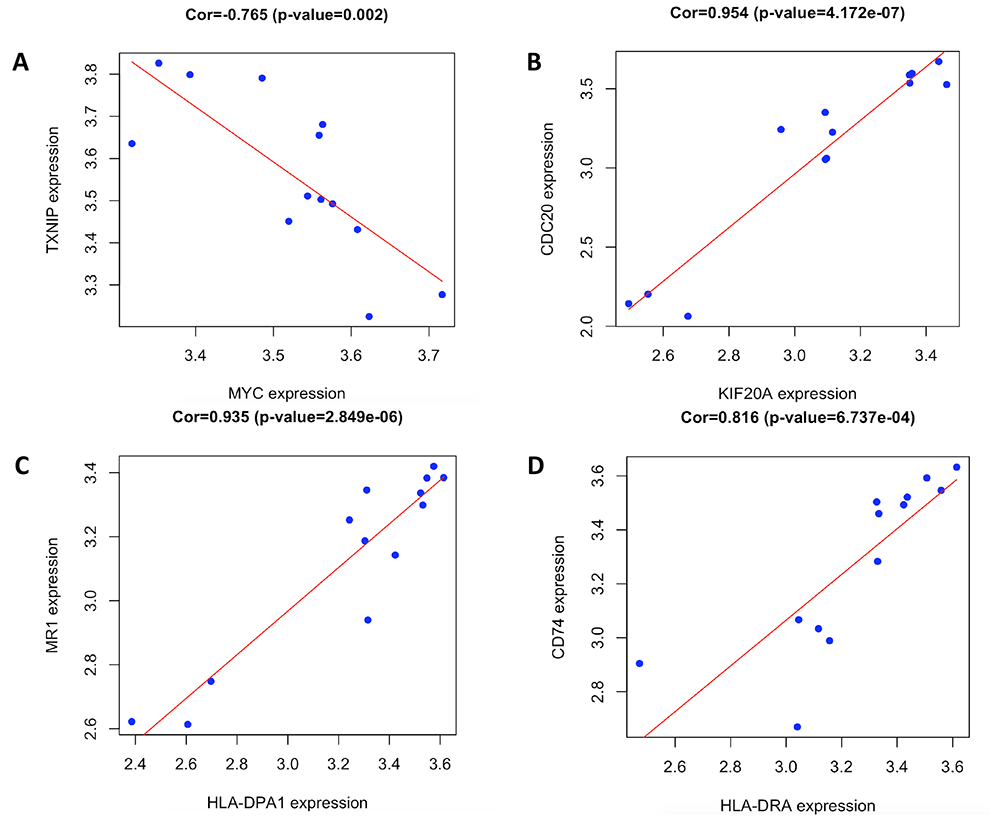

Supplement: Supplementary Figure 1 — The correlation profiles of (A) MYC and TXNIP; (B) KIF20A and CDC20; (C) HLA-DPA1 and MR1; (D) HLA-DRA and CD74. [file Image_1.TIF]
